# Suppression of Peroxisomal Enzyme Activities and Cytochrome P450 4A Isozyme Expression by Congeneric Polybrominated and Polychlorinated Biphenyls

**DOI:** 10.1155/2007/15481

**Published:** 2007-09-18

**Authors:** Larry W. Robertson, Isabelle Berberian, Tim Borges, Li-Chuan Chen, Ching K. Chow, Howard P. Glauert, Johannes G. Filser, Helmut Thomas

**Affiliations:** ^1^Graduate Center for Toxicology, University of Kentucky, Funkhouser Building, Lexington, KY 40506-0054, USA; ^2^Department of Occupational and Environmental Health, College of Public Health, University of Iowa, 124 IREH 100 Oakdale Campus, Iowa City, IA 52242-5000, USA; ^3^Oak Ridge National Laboratory, Oak Ridge, TN 37830, USA; ^4^Graduate Center for Nutritional Sciences, University of Kentucky, Funkhouser Building, Lexington, KY 40506-0054, USA; ^5^GSF-National Research Center for Environment and Health, Institute of Toxicology, Ingolstädter Landstraße 1, Neuherberg 85716, Germany; ^6^Tranzyme Pharma Inc., 3001 12th Avenue North, Building Z5-3037, Sherbrooke, QC J1H 5N4, Canada

## Abstract

The purpose of this study was to determine the effects of PCBs and PBBs on peroxisome proliferator-activated receptor-α-(PPARα-) associated enzyme activities or protein levels. Male Sprague-Dawley rats were administered a single IP injection (150 *μ* mol/kg) of either 3,3′,4,4′-tetrabromobiphenyl, 3,3′,4,4′-tetrachlorobiphenyl, 3,3′,5,5′-tetrabromobiphenyl, 2′,3,3′,4,5-pentachlorobiphenyl, 3,3′,4,4′,5-pentachlorobiphenyl, 2,2′,3,3′,5,5′-hexachlorobiphenyl, or 3,3′,4,4′,5,5′-hexabromobiphenyl in corn oil (10 ml/kg). One week later, the activities of catalase, peroxisomal fatty acyl-CoA oxidase, and peroxisomal beta-oxidation as well as cytochrome P450 4A (CYP4A) protein content were determined in subcellular liver fractions. None of the peroxisomal enzyme activities were significantly increased by any of the halogenated biphenyl congeners tested. Except for minor (approx. 25%) increases in the total CYP4A content following treatment with 2,2′,3,3′,5,5′-hexachlorobiphenyl and 3,3′,5,5′-tetrabromobiphenyl, CYP4A protein contents were not increased by any treatment. The two Ah receptor agonists, 3,3′,4,4′-tetrabromobiphenyl and 3,3′,4,4′,5-pentachlorobiphenyl, significantly diminished the liver content of CYP4A proteins and activities of the peroxisomal enzymes studied. Since a range of congeners with different biologic and toxicologic activities were selected for this study, it may be concluded that the polyhalogenated biphenyls do not induce peroxisome proliferation in the male rat, but rather certain members of this class of compounds down regulate peroxisome-associated enzymes. Since PCBs and PBBs do not increase enzyme activities and expression of proteins associated with PPARα, these agents are therefore exerting their carcinogenic and promoting activities by some other mechanism.

## 1. INTRODUCTION

The administration of any of a diverse class of chemicals, including plasticizers, hypolipidemic drugs, and perfluorinated fatty acids, leads to the activation of the peroxisome proliferator-activated receptor-α (PPARα), the expression of peroxisomal and nonperoxisomal proteins, and upon chronic administration, the induction of hepatic tumors in rodents [1]. These chemicals, known as peroxisome proliferators or peroxisome proliferator-activated receptor-α (PPARα) agonists, strongly increase both the number of peroxisomes in the rodent liver and the relative volume of the liver occupied by these organelles. The specific peroxisomal enzymes enhanced include the enzymes of fatty acid beta-oxidation and the carnitine acyl transferases [2].

Peroxisome proliferation is also accompanied by an increase in enzymes which are not directly associated with the organelle, including the members of the CYP4A gene subfamily which catalyze lauric acid hydroxylation [3, 4]. The induction of cytochrome P450 4A (CYP4A) isozymes inevitably accompanies peroxisome proliferation in the rodent, an effect which has been seen with a broad range of peroxisome proliferators, and it has been suggested that CYP4A and peroxisomal enzymes are coordinately regulated [1].

The polyhalogenated biphenyls, PCBs, and PBBs, are other classes of hepatic carcinogens that have profound effect on gene expression. PBBs and PCBs possessing no ortho halogens are efficacious inducers of CYP1A and many other enzymes, and avidly bind the aryl hydrocarbon (Ah) receptor [5]. Ortho-para-substituted PBBs and PCBs activate the constitutive androstane receptor (CAR) and pregnane X receptor (PXR) and induce CYP2B and CYP3A enzymes [6–8]. Like the peroxisome proliferators, halogenated biphenyls are efficacious promoters of two-stage hepatocarcinogenesis [9, 10], and are active as liver carcinogens in chronic bioassays [10, 11].

One possible mechanism for the chronic activities of PCBs and PBBs is the acute action of these compounds on the enzymes associated with PPARα. Previously, Borlakoglu et al. [12] found that PCBs increased CYP4A-related lauric acid hydroxylation and CYP4A1 protein levels. We therefore hypothesized that PCBs and PBBs may influence the activation of PPARα and thus influence the enzyme activities or protein levels of PPARα-associated proteins. In the present study, the effects of several selected congeneric polyhalogenated biphenyls, representing different classes of enzyme inducers, on peroxisomal enzyme activities and CYP4A protein levels are reported.

## 2. MATERIALS AND METHOD

### 2.1. Chemicals

3,3′,4,4′-Tetrachlorobiphenyl (PCB-77), 2′,3,3′,4,5,-pentachlorobiphenyl (PCB-122), 3,3′,4,4′,5-pentachlorobiphenyl (PCB-126), 3,3′, 4,4′-tetrabromobiphenyl (PBB-77), 3,3′,5,5′-tetrabromobiphenyl (PBB-80), and 3,3′,4,4′,5,5,′-hexabromobiphenyl (PBB-169) were synthesized as previously described [13, 14]. 2,2′,3,3′,5,5′-Hexachlorobiphenyl (PCB-133) was prepared via a multistep synthesis involving the chlorination of 2,2′,5,5′-tetrachlorobenzidine with N-chlorosuccinimide and subsequent deamination with hypophosphorous acid, as described by Kubiczak et al. [15]. The synthetic polyhalogenated biphenyl congeners were purified by Florisil (Macherey-Nagel, Duren, Germany) and Alumina (Aluminiumoxid 90, Merck, Darmstadt, Germany) chromatography and recrystallization from methanol. Structural assignments were confirmed by nuclear magnetic resonance spectrometry and mass spectroscopy. The purity of the individual congeners was >97%.

### 2.2. Experimental design

48 male Sprague-Dawley rats (approx. 40 g) were purchased from Harlan Sprague Dawley (Indianapolis, Ind, USA). The rats were randomly distributed into different groups (6 rats per group) and were placed on a purified diet similar to the AIN-76A diet [16, 17], which consisted of corn starch, 32.5%; dextrose, 32.5%; vitamin-free casein, 20.0%; cellulose fiber, 5.0%; corn oil, 5.0%; AIN mineral mix, 3.5%; AIN vitamin mix, 1.0%; DL-methionine, 0.3%; choline bitartrate, 0.2%. All dietary components were purchased from Teklad (Madison, Wis, USA). After 1 month, each animal (average weight: 230 g) was administered a single IP injection of a congeneric polyhalogenated biphenyl (150 *μ*mol/kg) in corn oil (10 mI/kg) or vehicle alone. The route of administration (IP), chosen to maximize delivery of the xenobiotic and minimize the contamination of facilities, and the time course of the experiment have been routinely used for expression studies with halogenated biphenyls (please see, e.g., [8]). One week after the administration of the halogenated biphenyl, each rat was euthanized with pentobarbital (120 mg/kg, IP). The liver was excised and homogenized with an Ultra-Turrax homogenizer (Tekmar Co. Cincinnati, Ohio, USA) in 4 volumes of 1.15% KCl-potassium phosphate buffer (0.05 M; pH 7.4). Aliquots of the homogenate were used for the determination of protein [18], and the activities of catalase [19], peroxisomal beta-oxidation [20], and fatty acyl-CoA oxidase [21], essentially as described. Hepatic 10 000 x*g* supernatants were prepared [22] and used for cytochrome P450 analyses, as described below.

### 2.3. Western analyses

Equal volumes of liver 10 000  x*g* supernatants from all animals per treatment group were combined. Aliquots containing 50 *μ*g of protein per lane were subjected to 10% SDS-PAGE [23] and blotted onto nitrocellulose as described by Towbin et al. [24]. The Western blots were incubated with 1 *μ*g/ml of primary monoclonal mouse antibody followed by incubation with a sheep antimouse IgG-horseradish-peroxidase conjugate. Staining was performed with 4-chloro-l-naphthol in the presence of hydrogen peroxide. The monoclonal antibody “clo4” is diagnostic for rat liver cytochrome P450 isozymes CYP4Al, CYP4A2, and CYP4A3, and was raised and characterized as described previously [25, 26]. Quantitation of CYP4A isozymes was carried out by densitometric scanning using a CAMAG II TLC scanner with Cat software Version 3.05. Results are expressed as integrated absorption units per 50 *μ*g 10 000  x*g* supernatant protein.

### 2.4. Statistical analyses

Data were analyzed by one-way analysis of variance and Dunnett's multiple comparison test [27].

## 3. RESULTS AND DISCUSSION

The activities of the enzymes catalase and fatty acyl-CoA oxidase, which are located within or attached to the membrane of the peroxisome, as well as the measurement of the flux through the peroxisomal beta-oxidation pathway are all highly specific peroxisomal activities. In male rats, fatty acyl-CoA oxidase and peroxisomal beta-oxidation increase 10–20-fold when potent peroxisome proliferators are administered, whereas catalase increases about 2-fold [2]. We have studied the regulation of these enzyme activities following application of structurally unrelated peroxisome proliferators and related xenobiotics [28–30]. None of the PCBs tested in the present study increased any of these activities (Table 1). Instead, the most potent Ah receptor agonist, PCB-126, significantly reduced the activities of fatty acyl-CoA oxidase and peroxisomal beta-oxidation, an observation which suggests that these halogenated biphenyls may diminish the liver's ability to break down long-chain fatty acids. Indeed, these compounds cause the concentration of neutral lipids within the liver of rats (fatty liver) (please see below). PCB-126 also lowered catalase activity. PCB-133 was the only PCB used to increase total CYP4A protein: to 123% of the control value as evidenced by immunoblot analysis. The other PCBs displayed either no effect (PCB-77), or slightly (PCB-126) or markedly (PCB-122) reduced total hepatic CYP4A protein levels.

PBBs also lowered the activities of hepatic peroxisomal enzymes (Table 2). The two PBBs that are “co-planar” and potent Ah receptor agonists, PBB-77 and PBB-169, both decreased peroxisomal beta-oxidation and FAO activity. PBB-77 also lowered hepatic catalase activity. PBB-80 did not affect any of the peroxisomal enzymes. As far as the effects of selected PBBs on hepatic CYP4A protein levels are concerned, only PBB-80 appeared to slightly increase CYP4A protein (to 126% of control), whereas PBB-77 and PBB-169 substantially diminished total CYP4A protein.

Previous studies also found that peroxisomal β-oxidation, catalase, and CYP4A were significantly suppressed in the male rat following the administration of PCB-126 [31–33]. The present study confirms these findings and extends it to other PCBs and PBBs (PCB-122, PBB-77, PBB-169). In contrast, Borlakoglu et al. [12] found an increase in CYP4A1 content in the livers of neonates which had been exposed to PCBs via lactational transfer, although, in the same study, the activity of catalase was unchanged, while peroxisomal beta-oxidation and FAO activities were diminished. However, since a polyclonal antibody was used in an ELISA to quantify CYP4A1 protein, there are certain questions about the specificity of the antibody and the resulting quantitation. In the present study, PCB-133 increased CYP4A protein levels, while not affecting FAO and catalase activities.

Peroxisome proliferators lower serum and liver lipids [34], whereas polyhalogenated biphenyls, particularly the Ah receptor agonists, cause an increase in the neutral lipid content of the liver (i.e., fatty liver) [35, 36]. The results of this investigation indicate that apparently neither the investigated compounds themselves nor the accumulated hepatic lipids were able to induce peroxisomal beta-oxidation and increase CYP4A isozyme expression. Instead, the suppression of peroxisomal beta-oxidation and reduction of CYP4A-mediated fatty acid omega-oxidation by certain polyhalogenated biphenyls (Ah receptor agonists) may be responsible to a considerable extent for the accumulation of hepatic lipids.

The reduction in peroxisomal beta-oxidation is not likely due to an inhibition of the individual enzyme activities by congeneric polyhalogenated biphenyls or metabolites, but rather due to a decrease in the amounts of these proteins, as was demonstrated for CYP4A. The relatively short half-life of peroxisomal enzymes of 1.5 days [37] leaves open the possibilities that either certain polyhalogenated biphenyls function to downregulate the expression of these proteins or, alternatively, their toxic action limits the ability of the cell to synthesize new protein, or protein catabolism is increased.

These findings imply that the mechanism by which PCBs and PBBs induce and promote hepatic tumors is different from that of peroxisome proliferators. The induction of hepatic tumors by peroxisome proliferators is dependent on the presence of PPARα [38]. Since PCBs and PBBs are not known to alter the expression of PPARα itself, the absence of an increase in the activities and levels of proteins associated with PPARα indicates that these agents are exerting their carcinogenic and promoting activities by some other mechanism. Other mechanisms by which PCBs and PBBs may exert their carcinogenic effects include altering other signal transduction pathways, increasing oxidative stress, influencing vitamin A metabolism, and inhibiting metabolic cooperation [9, 10].

In summary, the selected polyhalogenated biphenyls in the present study did not increase the enzyme activities associated with peroxisomal beta-oxidation or the CYP4A protein content. In fact, one group of more acutely toxic congeners (the Ah receptor agonists) significantly decreased the activities of catalase, fatty acyl-CoA oxidase, peroxisomal beta-oxidation, and CYP4A content. Based on a range of congeners with differing biologic properties tested, one may conclude that the polyhalogenated biphenyls do not induce peroxisome proliferation in the mature male rat.

## Figures and Tables

**Table 1:** Effect of PCBs on PPARα-related enzyme activities and protein levels.

Treatment	Catalase (units/mg/min)	FAO (nmol/mg/min)	Peroxisomal *β*-oxidation (nmol/mg/min)	Total CYP4A ${\text{(densitometry units)}}^{\text{a}}$

Control	418±96	2.73±0.43	2.93± 0.43	2354
PCB-77	355±75	2.66± 0.27	2.28 ±0.22	2301
PCB-122	411±47	2.58± 0.22	2.96± 0.62	1562
PCB-126	179±42 ^*^	0.69± 0.20 ^*^	1.62± 0.42 ^*^	1902
PCB-133	412±62	2.73± 0.29	2.61± 0.69	2898

Values are means ± SEM. Values significantly different from the control value are labeled with an asterisk (*), P<.05.

^a^Equal volumes of liver 10 000  x*g* supernatants from all animals per treatment group were combined; values represent the densitometric tracing from one lane.

**Table 2:** Effect of PCBs on PPARα-related enzyme activities and protein levels.

Treatment	Catalase (units/mg/min)	FAO (nmol/mg/min)	Peroxisomal *β*-oxidation (nmol/mg/min)	Total CYP4A ${\text{(densitometry units)}}^{\text{a}}$

Control	418±96	2.73±0.43	2.93± 0.43	1970
PBB-77	302±73 ^*^	0.67± 0.32 ^*^	1.34±0.34 ^*^	1042
PBB-80	429±81	3.10± 0.30	2.31± 0.46	2473
PBB-169	380±33	1.89± 0.23 ^*^	1.89± 0.23 ^*^	927

Values are means ± SEM. Values significantly different from the control value are labeled with an asterisk (*), P<.05.

^a^Equal volumes of liver 10 000  x*g* supernatants from all animals per treatment group were combined; values represent the densitometric tracing from one lane.
